# Supplementary material for: Technical evaluation and usability of a quantitative G6PD POC test in cord blood: a mixed-methods study in a low-resource setting
Source: BMJ Open. 2022 Dec 13;12(12):e066529. doi: 10.1136/bmjopen-2022-066529 (PMC9748916; doi:10.1136/bmjopen-2022-066529)
Supplement: Supplementary data [file bmjopen-2022-066529supp002.pdf]

Quantitative G6PD point-of-care test can be used reliably on cord blood to identify male and female newborns at increased risk of neonatal hyperbilirubinaemia: a mixed method study

Supplementary Figures and Tables

S Table 1. G6PD enzymatic activity (IU/gHb) of cord blood by spectrophotometry according to genotype

G6PD genotype	N	Mean	Std. Deviation	Minimum	Maximum
Hemizygote	26	1.64	0.65	0.09	3.32
Homozygote	3	1.66	0.43	1.38	2.16
Heterozygote	34	8.55	2.97	3.54	18.89
WT	262	13.62	2.02	8.01	26.32
Total	325	12.02	4.14	0.09	26.32

S Table 2. G6PD enzymatic activity (IU/gHb) by Biosensor according to genotype

G6PD genotype	N	Mean	Std. Deviation	Minimum	Maximum
Hemizygote	26	2.87	0.81	1.4	4.6
Homozygote	3	2.70	1.23	1.8	4.1
Heterozygote	34	9.50	3.47	4.0	18.6
WT	244	14.46	2.72	8.1	20.0
Total	307	12.82	4.47	1.4	20.0

S Figure 1. Absolute difference in G6PD activity detected by Biosensor as compared to spectrophotometry over time (only MKT clinic)

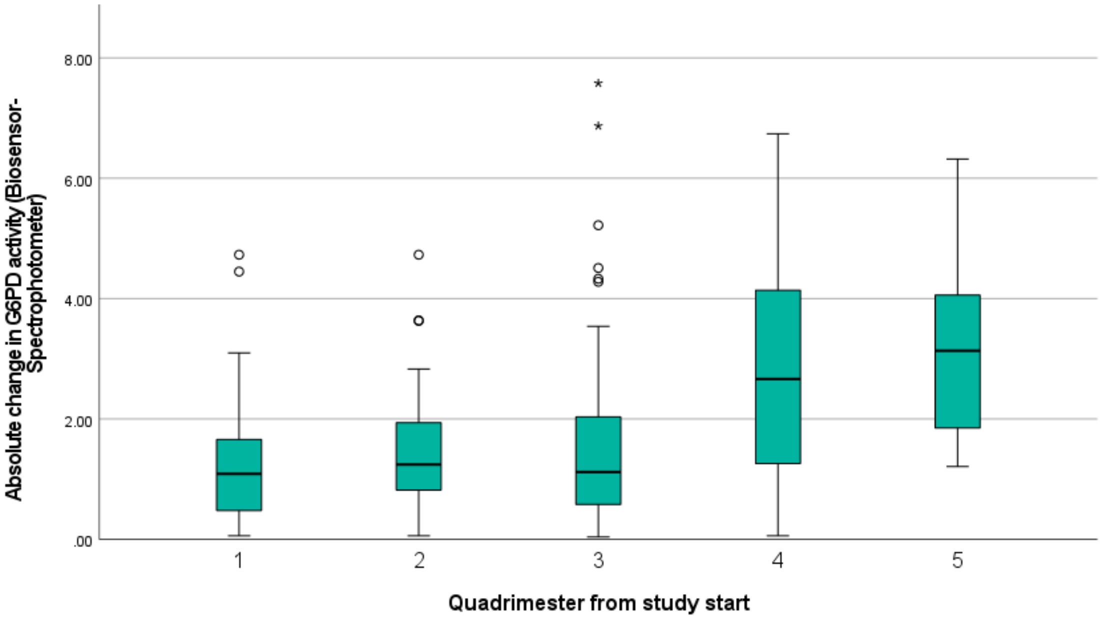

S Table 3. Characteristics of samples misclassified by Biosensor

Clinic	Year	EGA	Sex	Reference G6PD (IU/gHb)	Reference Hb (g/dL)	Percent activity of reference (%)	Reference phenotype	Biosensor G6PD (IU/gHb)	Biosensor Hb (g/dL)	Percent activity of Biosensor (%)	Percent activity of reference (%)	Biosensor phenotype	G6PD genotype Mahidol	Retics (%)	WBC (10^3/ uL)
MKT	2020	42	F	4.4	15	33	INT	4.3	15.2	30	32	DEF	Heterozygote	1.5	13.6
MKT	2021	40	F	7.1	15.8	54	INT	12.6	16.3	88	95	NOR	Heterozygote	1.3	20.2
MKT	2021	41	F	7.7	14.1	58	INT	12.5	11.5	87	94	NOR	Heterozygote	ND	20.6
MKT	2021	39	F	8.0	15.3	60	INT	10.9	11.7	76	82	NOR	WT	2.3	19.3
MKT	2021	39	F	8.2	14.3	62	INT	4	15.7	28	30	DEF	Heterozygote	2.2	21.1
MKT	2021	39	F	8.5	14.1	64	INT	11.8	14.1	82	89	NOR	Heterozygote	ND	ND
WPA	2021	39	F	9.4	13.3	71	NOR	9.8	13.7	68	74	INT	Heterozygote	1.6	13.8
WPA	2021	38	F	10.2	14.8	77	NOR	7.5	15.3	52	56	INT	Heterozygote	2.1	11.6
WPA	2021	39	F	10.9	15.6	82	NOR	8.8	16.7	61	66	INT	WT	1.6	12.5
MKT	2020	38	F	11.4	16.7	86	NOR	9.3	18.8	65	70	INT	WT	4.8	7.1
WPA	2021	39	F	11.7	14.3	88	NOR	8.1	16.5	56	61	INT	WT	1.8	11.1
WPA	2020	39	F	11.8	15.8	89	NOR	9.8	15.3	68	74	INT	WT	1.9	14.3
MKT	2020	39	F	12.1	12.6	91	NOR	9	13.2	63	68	INT	WT	3.9	14.4
MKT	2021	40	F	14.2	14	107	NOR	9.7	16.3	67	73	INT	WT	1.7	11.3
MKT	2021	37	F	15.1	11.2	113	NOR	8.2	12.1	57	62	INT	WT	3.7	15.5

S Table 4. Phototherapy treatment in newborns with EGA≥38 weeks with different G6PD phenotypes

G6PD phenotype by spectrophotometry	PT	No PT	% PT	RR	95%CI	P_{Fisher}
Deficient	8	20	28.6	4.9	2.3-10.5	<0.001
Intermediate	3	17	15.0	2.6	0.8-8.1	0.13
Normal	15	242	5.8			reference
G6PD phenotype by Biosensor						
Deficient	9	21	30.0	5.4	2.5-11.6	<0.001
Intermediate	2	20	9.1	1.7	0.4-6.8	0.49
Normal	13	223	5.5			reference
